# Mechanical behavior of hot extruded aluminum 6082 chip

**DOI:** 10.1038/s41598-024-55151-0

**Published:** 2024-03-16

**Authors:** Seif El Din Mahmoud, Ramadan El-Gamasy, Ayman A. Abd El-Wahab

**Affiliations:** Department of Design and Production Engineering, Ain Shams University, Cairo, Egypt

**Keywords:** Aluminum recycling, Aluminum chips, Hot extrusion, Mechanical behavior, Microstructure, Solid-state recycling, Materials science, Mechanical engineering

## Abstract

In a bid to address the energy-intensive nature of primary aluminum production, this study explores the solid-state recycling of aluminum alloy 6082 chips through direct hot extrusion. Compacted at room temperature, chips were extruded at temperatures (350, 425, and 500 °C) and reduction ratios (6, 8.5, and 11) to optimize mechanical properties. Extensive analyses, including ANOVA and linear regressions, of strength, density, and microstructure revealed significant influences of those extrusion parameters. Optimizing these parameters within the study's aforementioned working ranges can impact the recycled material's strength; with a 36% reduction ratio increase and 20% temperature increase modestly reducing ultimate strength (2%), while a 20% temperature increase alone lowers yield strength more noticeably (9%). These findings highlight the potential for enhanced recycling and sustainable manufacturing.

## Introduction

The production of primary aluminum is a highly energy-intensive process in primary metal production. It demands approximately 10 times more energy than steel production, making it crucial to explore energy-efficient methods for aluminum production^1^. The global aluminum production in 2010 was 41.2 million tons, with a recycling rate of 20%, expected to increase to 50% by 2030^2^. Recycling of aluminum can significantly reduce the amount of energy required for production, especially through the re-melting of aluminum alloy scrap. However, recycling aluminum machining chips, a type of aluminum scrap, is challenging due to their high surface to volume ratio, which intensifies oxidation. Hot extrusion offers advantages over cold extrusion for aluminum chips due to its high temperatures breaking down these oxides and promoting bonding, making it a superior choice for this material. In light of these challenges, solid-state recycling processes like hot extrusion have emerged as promising alternatives, not only in overcoming these hurdles but also in improving the energy balance of aluminum production^1^.

Recycling is a vital strategy to address the environmental impact of waste generation. However, there are concerns that increased recycling efforts may lead to a rebound effect, wherein individuals' increased recycling efforts may result in higher resource consumption^3^. Efficient recycling methods that minimize material loss and enhance the mechanical properties of recycled aluminum are necessary to mitigate such effects. Among various aluminum scraps, machining chips from semi-finished products are particularly difficult to recycle due to their elongated spiral shape and low apparent density, making handling and transportation inconvenient. Conventional recycling processes are characterized by high energy consumption, operating costs, and material losses, limiting the recycling rate to less than 55% for aluminum scraps^4^. Thus, the development of more efficient and resource-saving recycling techniques is crucial for increasing the sustainability of aluminum production.

Direct conversion of aluminum alloy machining chips into finished or semi-finished products through hot extrusion has shown promise as an energy-efficient solid-state recycling method. In this process, the chips are compacted into billets and extruded using a conventional hot extrusion press. Proper extrusion die design is crucial to breaking the oxide layers on the chips' surfaces, enabling contact between pure metal surfaces and improving mechanical properties. The extrusion parameters, such as the extrusion ratio (R) and temperature, significantly affect the resulting mechanical properties of the chip-based extrudates^1^. It is essential to optimize the hot extrusion process to achieve high-quality chip-based extrudates comparable to those obtained from extruded cast material.

Several research studies have delved into hot extrusion and solid-state recycling of aluminum alloys, offering crucial insights into process optimization and mechanical property enhancement. Studies on extrusion parameters have highlighted their significant influence on the resulting material properties. Tekkaya et al.^1,5^ demonstrated the potential of hot profile extrusion for AA6060, achieving a 15% increase in ultimate tensile strength with a reduction ratio of 34.2. Chiba et al.^6^ emphasized the importance of parameter selection in cold extrusion and rolling of AC4CH chips. Güley et al.^2^ explored the impact of die design on welding quality during hot extrusion of AA6060 chips, providing valuable guidance for process control. Research on process optimization has yielded promising results for various aluminum alloys. Zuo et al.^7^ pinpointed 400 °C as the optimal extrusion temperature for AA6063, while Zhang et al.^8^ identified 250 °C as the ideal preheating temperature for AA5083. Wagdy^9^ validated an eco-friendly hot extrusion method for AA2011 using statistical analysis, aligning with sustainability goals. Explorations of alternative techniques have also shown potential. El-Habbas et al.^10^ proposed a two-step approach combining hot extrusion with equal channel angular pressing (ECAP), demonstrating effective microstructure refinement and property enhancement. While studies on other alloys can offer valuable insights, Jian-Yih Wang et al.^11^ investigated solid-state recycling of Magnesium Alloy AZ91D through hot extrusion, providing a broader understanding of the process's applicability across materials. Collectively, these studies underscore the importance of parameter optimization, process tailoring, and innovative techniques in advancing solid-state recycling of aluminum alloys.

This paper aims to advance the understanding of the hot extrusion process for recycling chips of aluminum alloy 6082 and investigate its mechanical behavior, building upon previous research findings. The investigation of the mechanical behavior of recycled aluminum alloy 6082 holds significant potential for developing energy-efficient and sustainable recycling processes, which align with environmentally friendly manufacturing practices.

After conducting a thorough review of several published papers^1,2,5–11^, it was found that a number of parameters had been studied and correlated with the properties of recycled materials. However, it was also found that the interaction between extrusion temperature and extrusion reduction ratio and its impact on the mechanical behavior of solid-state recycled samples in comparison to conventionally recycled ones have been rarely explored. In light of this gap in the literature, this study has chosen to focus on aluminum alloy 6082 chips and employ direct hot extrusion as the recycling method.

The selection of Aluminum 6082 as the alloy for this study was made due to its widespread commercial availability and the diverse range of applications it can be used for. This alloy is commonly used in a variety of industries, such as construction, transportation, and manufacturing. Additionally, it is relatively easy to obtain and thus was deemed a practical choice for this research. Furthermore, Aluminum 6082 has a good combination of properties such as strength, corrosion resistance, weldability and machinability, so it’s a suitable candidate for many engineering applications.

The primary emphasis of this research will be on investigating the combined influence of extrusion temperature and ratio, as both parameters play crucial roles in determining the mechanical properties of the recycled aluminum alloy. By examining these aspects simultaneously, this study aims to contribute valuable insights into optimizing the hot extrusion process for efficient and effective recycling of aluminum alloy 6082 chips.

## Methodology

### Materials

The raw material used for chip formation in this study was an Aluminum 6082 hollow stock with inner diameter of 25 mm and outer diameter of 50mm. It was purchased from a local supplier, and the chemical composition of the material was found to be as indicated in Table 1.

**Table 1:** Chemical compositions of the Aluminum alloy to be recycled (parent material) and standard Aluminum alloy.

Alloy	Si	Fe	Cu	Mn	Mg	Zn
6082*	0.96	0.13	0.07	0.48	0.79	0.01
6082**	0.7–1.3	0–0.5	0–0.1	0.4–1	0.6–1.2	0–0.2

*Chemical composition measured using an optical emission spectrometer (OES).

**Standard chemical composition as per to The Aluminum Association registration records^12^.

### Chip formation

According to the ASM "American Society for Metals" Handbook^13^, Aluminum alloy 6082 has a machinability rating of C. This rating suggests using High-speed steel turning tools with tool angles and at cutting parameters indicated in Table 2.

**Table 2:** Tool angles (A) and cutting parameters (B) for HSS turning tools.

(A)	
Rake angle	20–30°
Clearance angle	6–10°

(B)	
Depth of cut	0.4–6.4 mm
Feed rate	0.15–2.0 mm/rev
Speed	≤ 300 m/min

**Figure 1:**
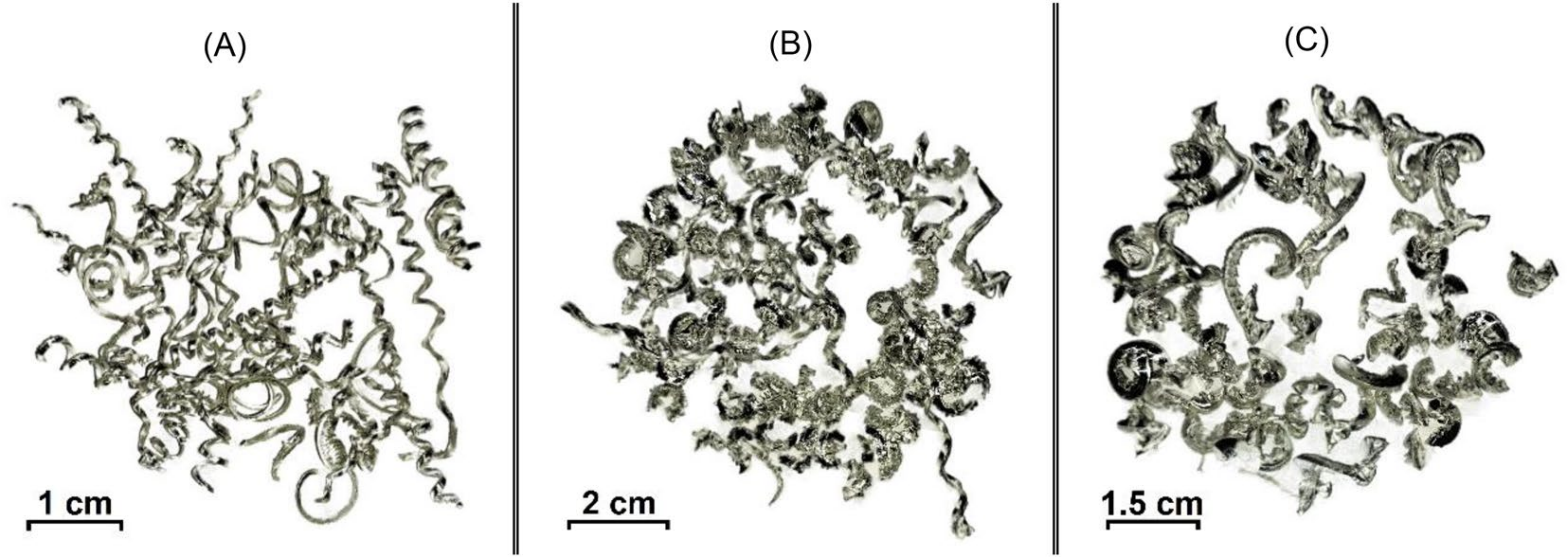
Aluminum alloy 6082 chips formed via machine turning process at different depths of cut 'a'—(**A**) a = 0.5 mm, continuous chips; (**B**) a = 1 mm, curled or easily broken chips; (**C**) a = 1.5 mm, very small broken chips.

After utilizing the tool design and cutting parameters mentioned earlier, the aluminum hollow stock underwent turning at a consistent speed and feed rate. This process was carried out at three distinct depths of cut: 0.5 mm, 1 mm, and 1.5 mm, resulting in chips of different lengths and thicknesses. These variations are visually represented in Fig. 1. Later on, the depth of cut was set at a constant value of 1mm, as it represented the average value.

### Compaction

**Figure 2:**
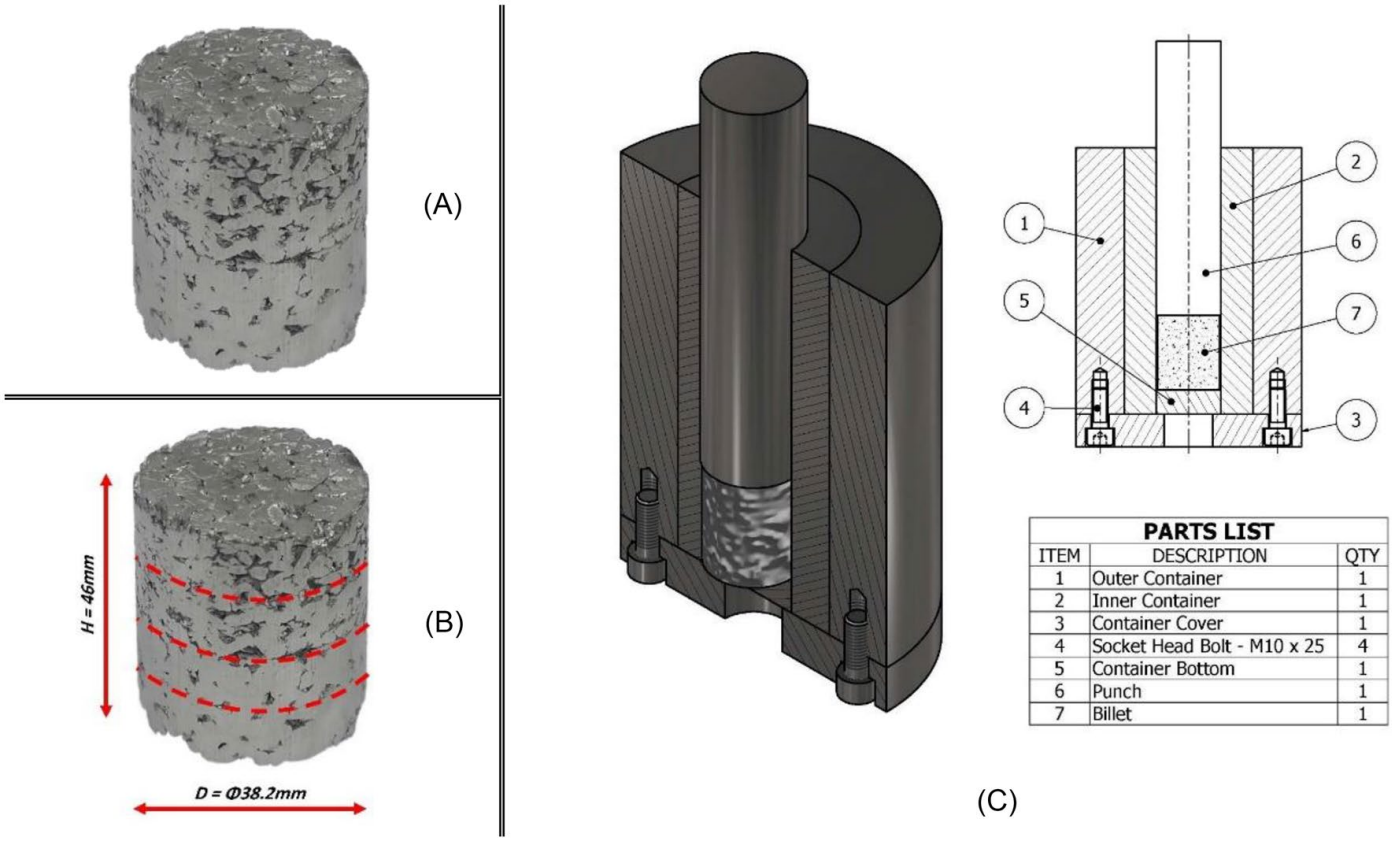
Compaction of Aluminum chips—(**A**) Compacted billed; (**B**) Average dimensions for the nine billets compacted during this study; (**C**) Detailed schematic of the designed and manufactured compaction/extrusion die and container.

For the compaction process, a die and die container, shown in Fig. 2C, were designed and manufactured for both purposes of compaction and later hot extrusion process. The material selected for the manufacturing of the die and container was high-chrome tool steel.

For this study’s purposes, nine billets have been compacted at room temperature; each representing a unique combination of extrusion variable levels. The average weight, diameter, and height of these billets are approximately 125 g, 38.2 mm, and 46 mm, respectively. Additionally, the dashed lines visible in Fig. 2B represent the radial seams on the exterior, which are indicative of the incremental addition and compaction process used to achieve the final height of the billet.

### Extrusion

**Figure 3:**
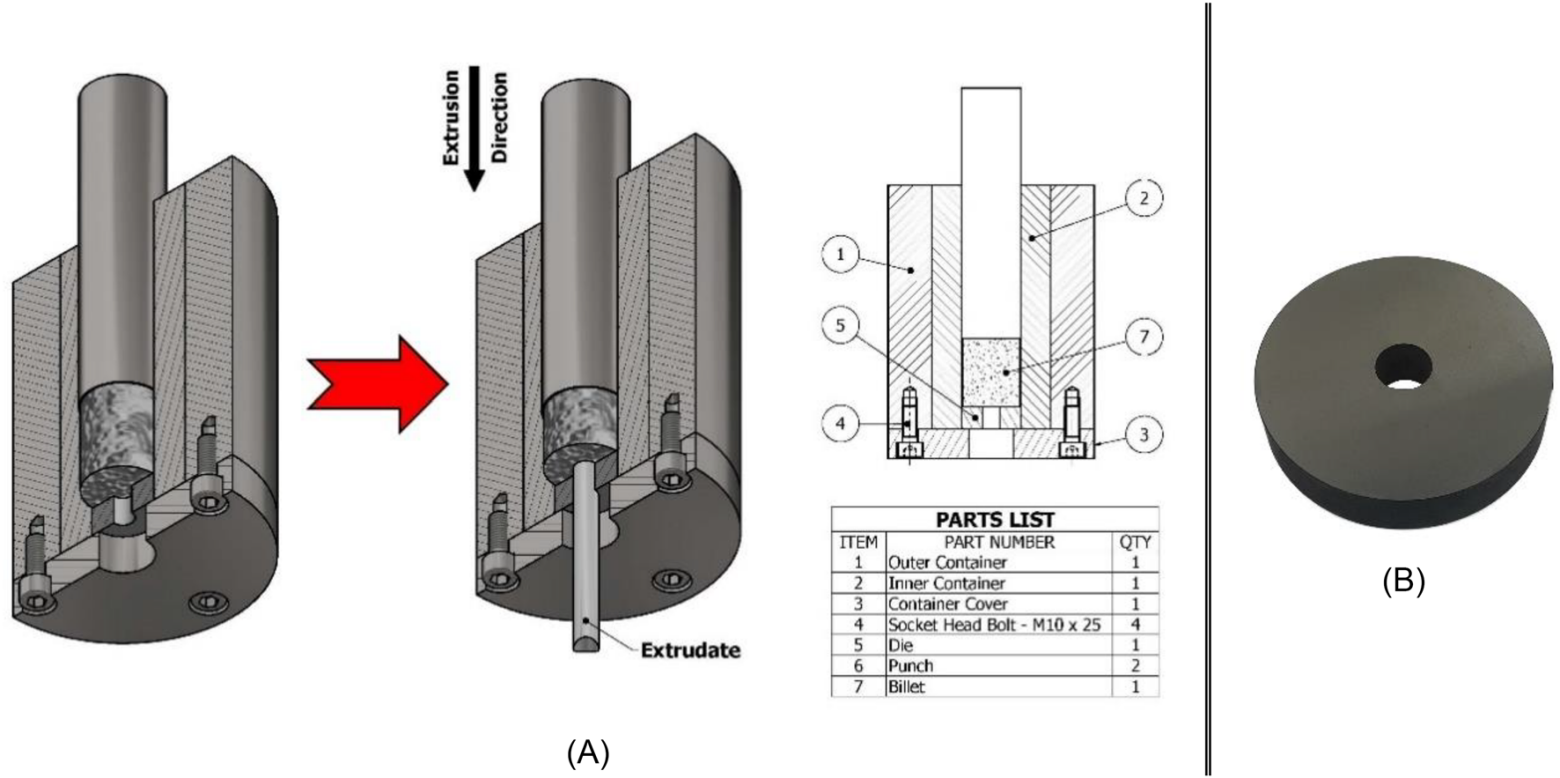
Direct hot extrusion of compacted aluminum chip billets—(**A**) Detailed schematic of the extrusion process; (**B**) High-chrome tool steel (BÖHLER W302) die for circular profile extrusion.

The type of metal formation process selected for the purpose of aluminum chip solid-state recycling was direct hot extrusion; shown in Fig. 3A. As previously stated, it was determined to vary two critical process parameters that significantly impact the final product's quality: the extrusion working temperature and the extrudate cross-sectional reduction ratio. Each parameter was explored at three different points, resulting in a 3 × 3 matrix of potential combinations (refer to Table 3), hence the amount compacted billets.

**Table 3:** Parametric conditions of each extrusion sample in terms of extrusion reduction ratio and extrusion working temperature.

	Sample #								
	1	2	3	4	5	6	8	9	10
Ratio	11	11	8.5	8.5	6	6	11	6	8.5
Temp. (°C)	350	500	350	500	350	500	425	425	425

The 6082 aluminum alloy has a melting temperature of 585 °C, and the recrystallization temperature of aluminum alloys may vary from 340 to 400 °C, so it was decided that the three points of work for the extrusion temperature parameter will be 350 °C, 500 °C, and their average value; 425 °C.

Knowing that as the reduction ratio increases, the area of the die cross-section decreases, leading to an increase in the force required to push the billet through the die. Another constraint to be faced was the direct relationship between the reduction ratio and the extrudate length. Considering these facts, while taking the capacity of the hydraulic press on hand, it was decided that the maximum ratio to work at will be 11, minimum ratio will be 6, and the third point will be their average value which is 8.5.

A prevalent issue known as "die swell" was discovered at the tip of the extrudate. This defect arises from the elastic properties of the extruded material, which causes it to return to its original shape and cross-section as it exits the die. Die swell can be triggered by insufficient lubrication or excessive friction between the extrudate's surface and the die opening, resulting in surface irregularities.

**Figure 4:**
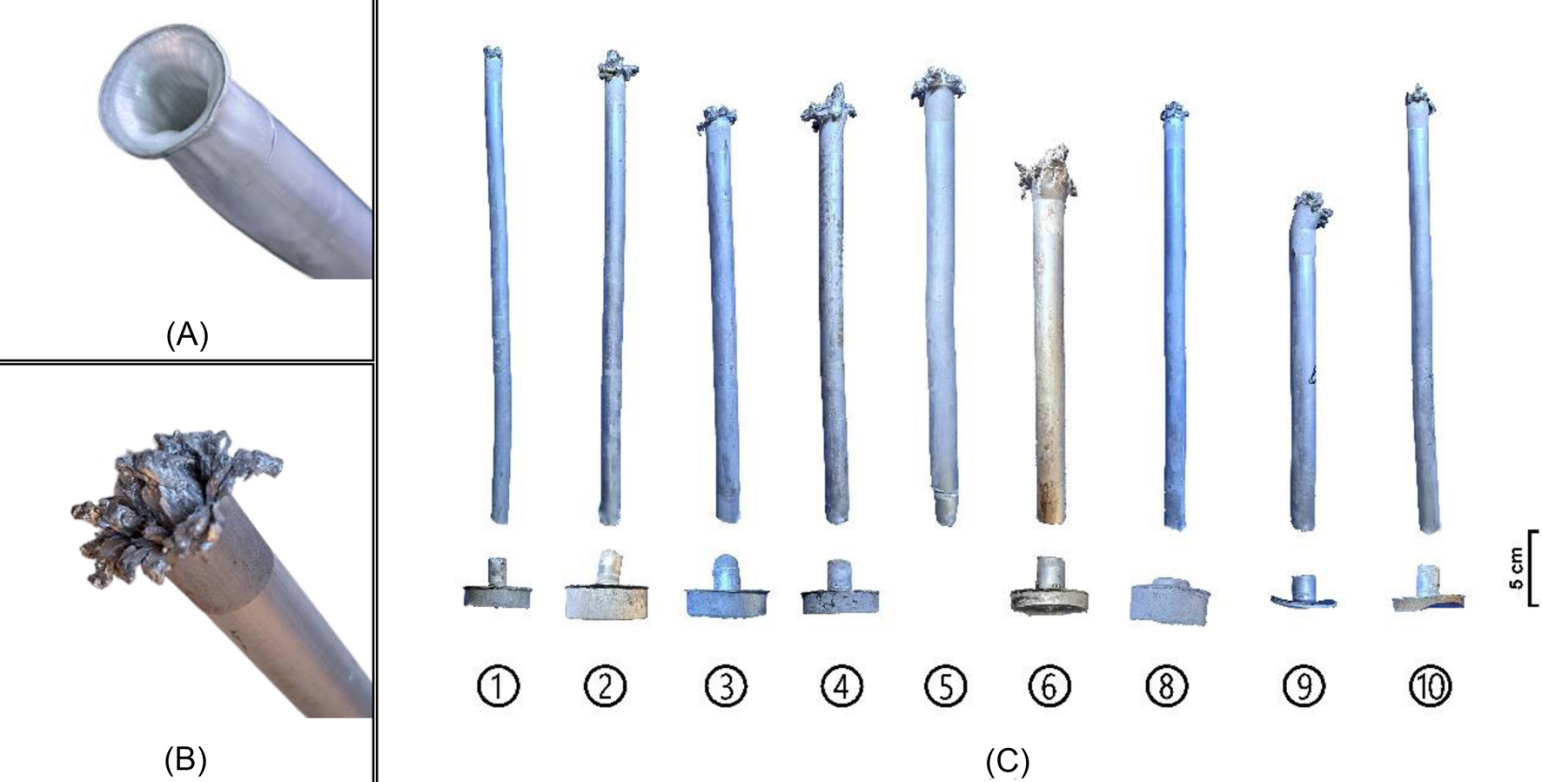
Hot extruded rods “Extrudates”—(**A**) Extrusion piping defect; (**B**) Die swell defect; (**C**) Extrudate rods after being separated from their dead-metal zones and labeled from 1 to 10. Each label represents a specific parametric combination of the extrusion conditions, except for No. 7, which was eliminated due to an experimental mishap.

While die swell is more commonly associated with polymer extrusion, it was also observed during the experiment of this study due to the elevated temperatures, relatively high reduction ratios, and lack of lubrication in the extrusion process. The presence of remnant voids between the material's particles from the compaction process likely contributed to this defect, resulting in a distinct flower-like appearance, as shown in Fig. 4B. Another possible reason to why this phenomenon occurred lack of shear action at extrudate tip at the beginning of the extrusion process.

Another common extrusion defect encountered during this study is extrusion piping or funneling, depicted in Fig. 4A. This issue is believed to have occurred due to overshooting the extrusion press ram displacement, neglecting the preset 1-cm safety distance. This oversight led to the extrusion of all the designated material inside the container, leaving behind no dead-metal zone, and posing a risk of damaging both the punch and extrusion die.

### Tensile samples making

**Figure 5:**
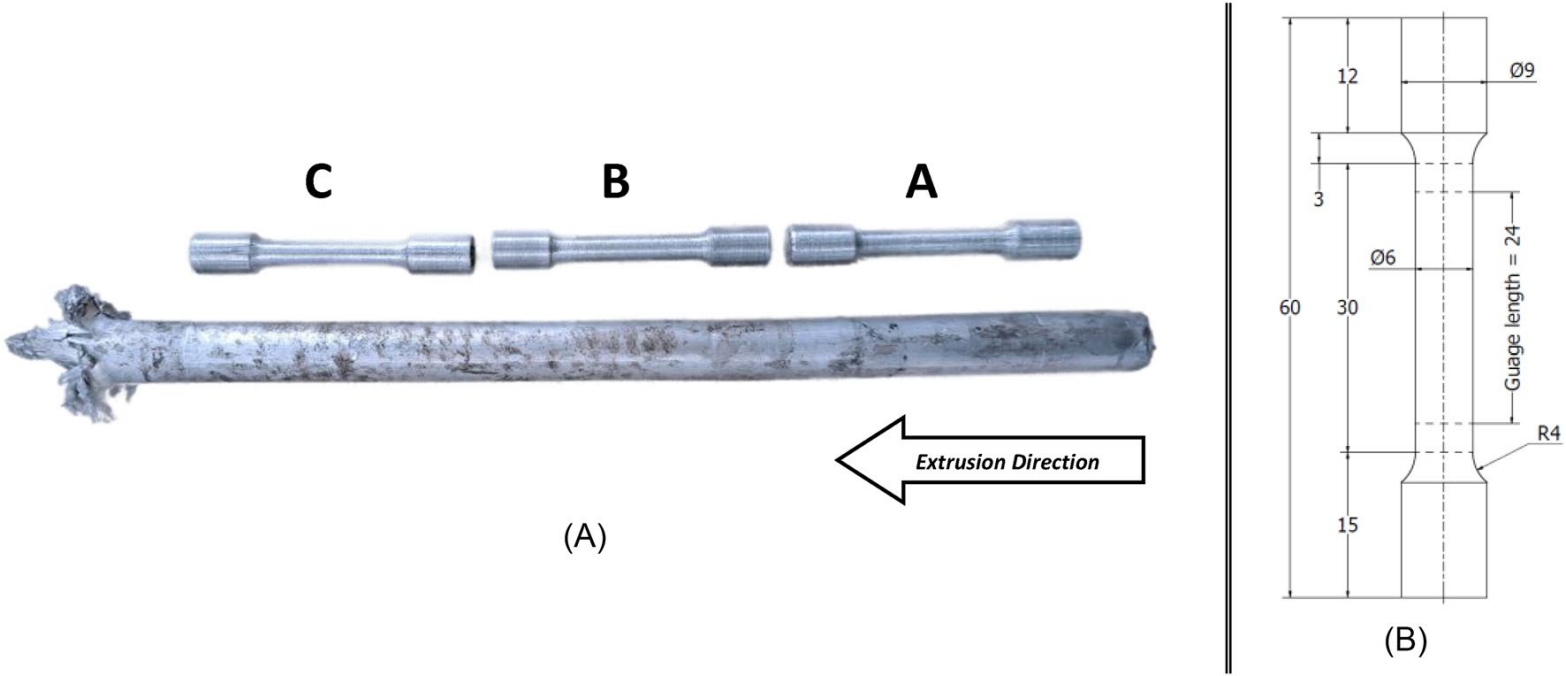
Tensile samples making—(**A**) Extrudate divided into three tensile samples and each given a letter; (**B**) Tensile sample manufactured as per ASTM E8/E8M, Sect. 6.6.1, specimen 3 [14].

Tensile specimens have been manufactured from the recycled extrudates on a center lathe turning machine according to ASTM E8/E8M. Figure 5 shows that each extrudate was divided into three sections; A, B and C, yielding three tensile specimens per extrudate. Each specimen was given a code (i.e. 3B), indicating the original extrudate, and the specimens position along the extrudate. The parent material hollow stock has also undergone work to make tensile specimens for testing under the same conditions the recycled specimens went through.

### Testing

#### Tensile test

After performing the test on all the specimens; 0A through 10C, using a universal Testing machine (Lloyd—300 kN), at a constant crosshead speed 2mm/min, a stress/strain curve is drawn for each specimen using 200 measured points (Tracking load, elongation, stress and percentage Strain). Strain was calculated by tracking the testing machine’s crosshead displacement and dividing the value by the specimen’s original gauge length.

#### Density

After conducting tensile testing on dog-bone specimens representing various extrusion conditions and sections of the extrudate rod, it was necessary to record the density of the specimens in order to assess variations and correlate them with the parameters being studied. This analysis was performed by hydrostatic weighing method using an analytical balance density determination kit (Adam Equipment PW 254—Max. Capacity: 250 g, Resolution: 0.0001 g).

#### Microstructure

Another way to compare between the parent material alloy and the recycled one is to study their microstructural properties; such as grain size and shape, grain boundaries intensity and percentage of voids. In order to conduct such comparison, the specimens had to be studied metallographically. In order to conduct that procedure, the metallographic mounts had to be immersed in electropolishing and/or microetching solutions first. Metallographic electropolishing is a process that uses electrical current to remove surface material and create a highly polished surface. Microetching is a chemical process used to selectively dissolve or remove certain regions of a metal sample, revealing the microstructure. It can be used to reveal specific microstructural features, such as grain boundaries, phases, and defects. The specifications for both processes are shown in Table 4.

**Table 4:** Specifications of the metallographic electropolishing and microetching processes.

	Electropolishing		Microetching	
Standard	ASTM E1558-09 (III-14)		ASTM E407-99 (Method 3)	
Solution formula	Water Ethanol 95%	Phosphoric acid 85%	HF HCL	HNO_3_ Water
Ratio (respectively)	25:38:40		2:3:5:190	
Voltage	50 VDC		–	
Immersion time	4–6 min		5–15 s	
Working temperature	80 °C		–	

## Results and discussion

### Tensile test

#### Results

**Figure 6:**
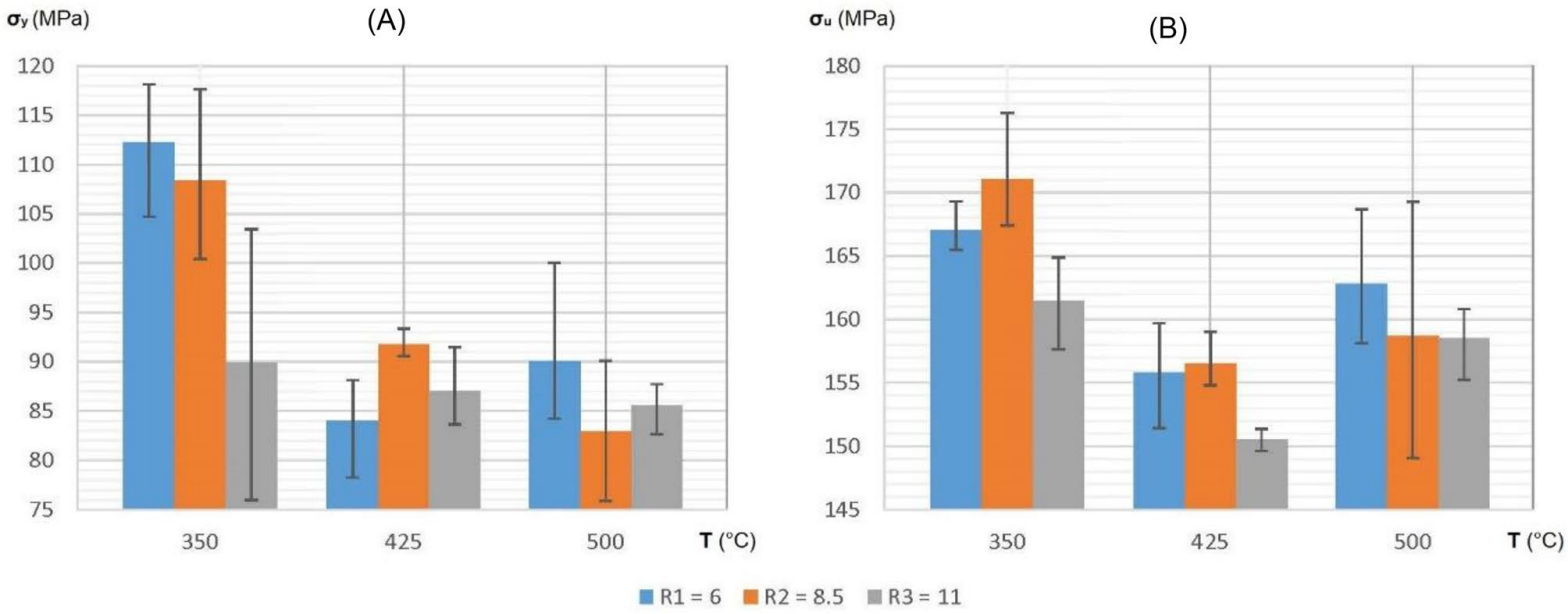
Histograms showing yield strength (**A**) and ultimate strength (**B**) of each tensile sample representing every combination between the values of extrusion temperature and reduction ratio.

**Figure 7:**
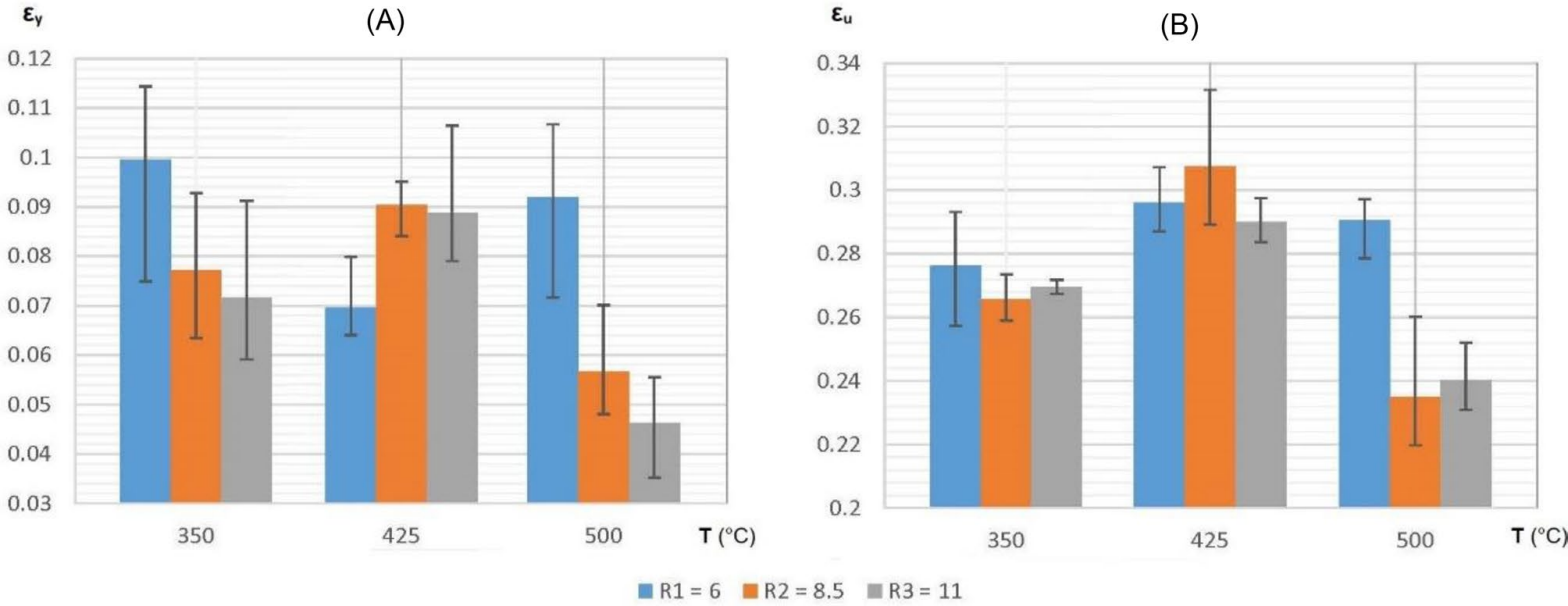
Histograms showing yield strain (**A**) and ultimate strain (**B**) of each tensile sample representing every combination between the values of extrusion temperature and reduction ratio.

In Figs. 6 and 7, the error bars per each bin on the histogram represent the variation between the mechanical properties (yield and ultimate strengths and strains, respectively) measured at zones A, B and C on each extrudate.

#### Analysis of variance (ANOVA)

This analysis section focuses on the influence of extrusion parameters on the mechanical properties of the recycled material. A full factorial design with three levels of reduction ratio and three levels of extrusion temperature was implemented. Individual and interaction effects between these two parameters on the recycled material's mechanical properties were evaluated using a two-way ANOVA model with interaction terms. Statistical significance was determined through F-tests comparing mean square ratios (MSRs) of rows, columns, and interactions with the F-critical value at 95% confidence. Additionally, P-values of each F-statistic was calculated to provide further insight on the observed effect is likely to be real (p < 0.05) or due to chance.

Based on the information presented in Fig. 6A and Table 5, it can be inferred with 95% confidence that the yield strength of the extruded material is notably influenced by the extrusion temperature (p < 0.05). However, the extrusion reduction ratio has a non-significant individual effect (p > 0.05). Nonetheless, there is a statistically significant interaction effect between temperature and reduction ratio (p < 0.05), indicating that the reduction ratio's impact depends on the temperature. This suggests that the combined effect of these factors is more than the sum of their individual effects.

**Table 5:** Analysis of Variance (ANOVA) in two-factor experiment to determine significance of extrusion reduction ratio and extrusion temperature individually and their interaction on the yield strength of the recycled material.

Sources of variation	SS “Sum of squares”	DF “Degrees of freedom”	MS “Mean square”	MSR (F-value)	P-value	Min. MSR*
Among columns (ratio)	${SS}_{c}=\frac{\sum {T}_{c}^{2}}{nr}-\frac{{T}^{2}}{N}=334$	${DF}_{c}=c-1=2$	${MS}_{c}=\frac{{SS}_{c}}{{DF}_{c}}=167$	${MSR}_{c}=\frac{{MS}_{c}}{{MS}_{e}}=3.07$	0.07126	F_0.05:2:18_ = 3.55
Among rows (Temp.)	${SS}_{r}=\frac{\sum {T}_{r}^{2}}{nc}-\frac{{T}^{2}}{N}=1664.8$	${DF}_{r}=r-1=2$	${MS}_{r}=\frac{{SS}_{r}}{{DF}_{r}}=832.4$	${MSR}_{r}=\frac{{MS}_{r}}{{MS}_{e}}=15.31$	0.00013	F_0.05:2:18_ = 3.55
Column-row interaction	${SS}_{cr}=\frac{\sum {T}_{cr}^{2}}{n}-\frac{{T}^{2}}{N}-{SS}_{c}-{SS}_{r}=688.7$	${{DF}_{cr}=DF}_{c}\times {DF}_{r}=4$	${MS}_{cr}=\frac{{SS}_{cr}}{{DF}_{cr}}=172.2$	${MSR}_{cr}=\frac{{MS}_{cr}}{{MS}_{e}}=3.17$	0.03887	F_0.05:4:18_ = 2.93
Residual (Error)	${SS}_{e}={SS}_{T}-{SS}_{c}-{SS}_{r}-{SS}_{cr}=978.6$	${DF}_{e}=N-cr=18$	${MS}_{e}=\frac{{SS}_{e}}{{DF}_{e}}=54.4$			
Total	${SS}_{T}=\Sigma {x}^{2}-\frac{{T}^{2}}{N}=3666.1$	${DF}_{T}=N-1=26$				

*Minimum F-value required for factors to be significant at 95% confidence on the F distribution curve.

**r:** No. of rows, **c:** No. of column, **N:** Total no. of tests, **n:** No. of tests per combination, **T (Σx):** Total of all test values, **Σx**^**2**^**:** Sum of square of all values, **T**_**c**_**:** Total for each column, **T**_**r**_**:** Total for each row, **T**_**cr**_**:** Total for each column-row combination.

By utilizing the data in Fig. 6B, and following the same procedure (ANOVA) done in Table 5, the F-values of columns, rows, and interaction turn out to be 3.55, 15.04 and 0.88, respectively. The P-values calculated from these F-statistics are 0.05016, 0.00014 and 0.43189. These values provide, with 95% confidence, that the ultimate strength of the extruded material is influenced by both the extrusion temperature (p < 0.05) and reduction ratio (p = 0.05), but there appears to be no interaction effect between them (p > 0.05). This suggests that the effect of one factor on the dependent variable is not dependent on the other factor, and their impact is consistent across all levels of the other factor. As a result, the two factors are operating independently, and their combined effect on the dependent variable is simply the sum of their individual effects.

#### Linear multiple regression analysis

Another method to analyze the relationship between strength of the extrudate “dependent variable” and extrusion temperature and reduction ratio “independent variables” is linear multiple regression (LMR) analysis. The goal of the analysis is to create a linear equation that can be used to predict the value of the dependent variable based on the values of the independent variables.

In linear multiple regression analysis, the relationship between the dependent variable and the independent variables is assumed to be linear. The analysis estimates the coefficients of the linear equation using a set of observed data points, with the goal of minimizing the differences (error) between the predicted values and the actual values of the dependent variable.

The linear equation used in multiple regression analysis is often represented as:

$${\upsigma }_{{\text{th}}}={\upbeta }_{0}+{\upbeta }_{1}{\text{T}}+{\upbeta }_{2}{\text{R}}+\upvarepsilon$$

where: σ_th_: Theoretical Yield or Ultimate Strength, T: Extrusion Temperature, R: Extrusion Ratio, ε: Error term, Β_0_: Intercept on the σ axis, Β_1_: Regression coefficient for T, Β_2_: Regression coefficient for R.

In order to validate the data analysis, correlation coefficients (R_σ,T_ and R_σ,R_) and coefficient of determination (R^2^) have to be calculated first. Correlation coefficients measures the strength and direction of the linear relationship between the dependent variable and each of the independent variables, while the coefficient of determination is a measure of the proportion of the total variation in the dependent variable that is explained by the independent variables in the regression model.

**Figure 8:**
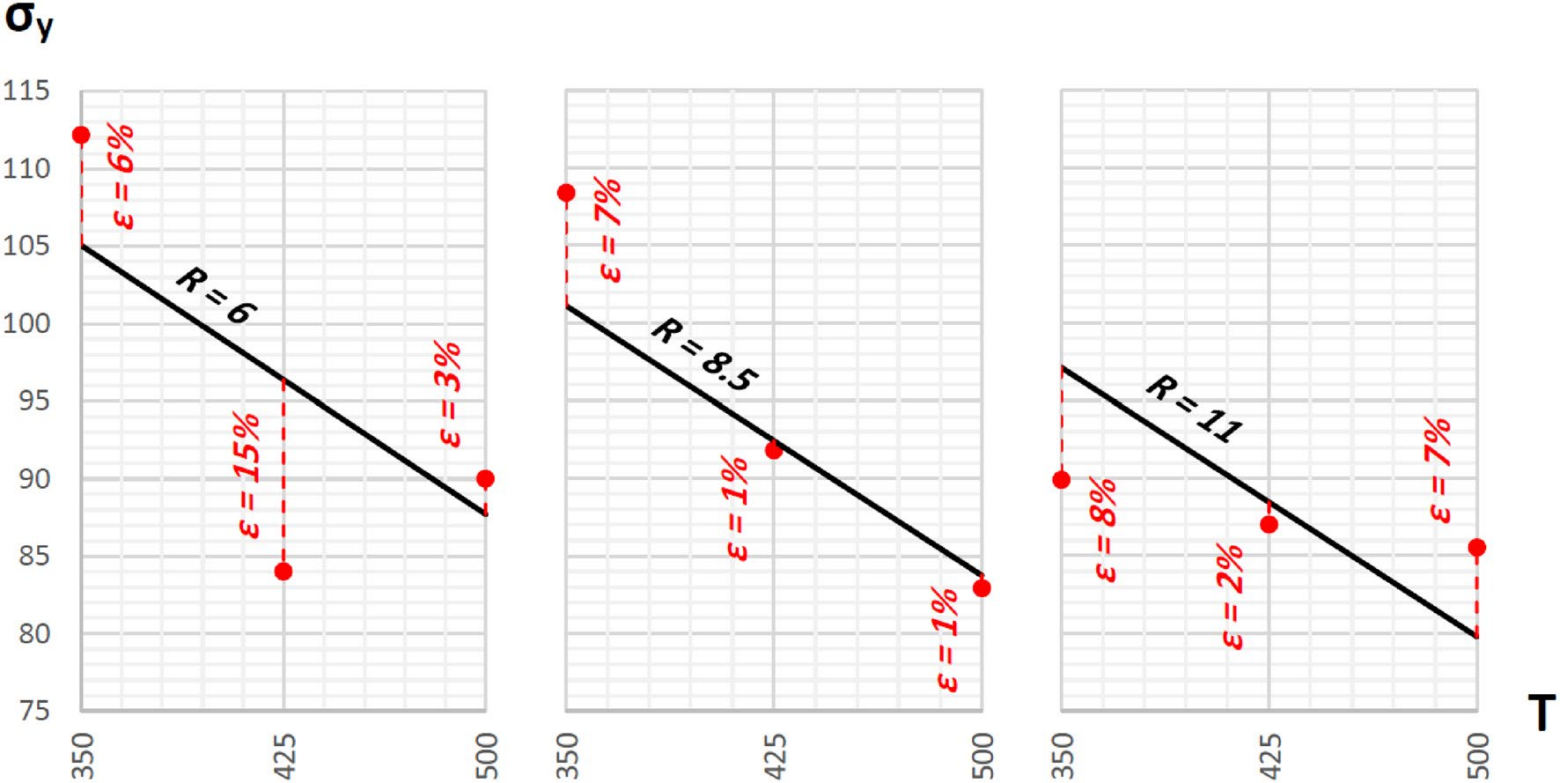
Regression lines representing theoretical yield strengths plotted against actual measured points and the errors between them (red dashed lines).

**Figure 9:**
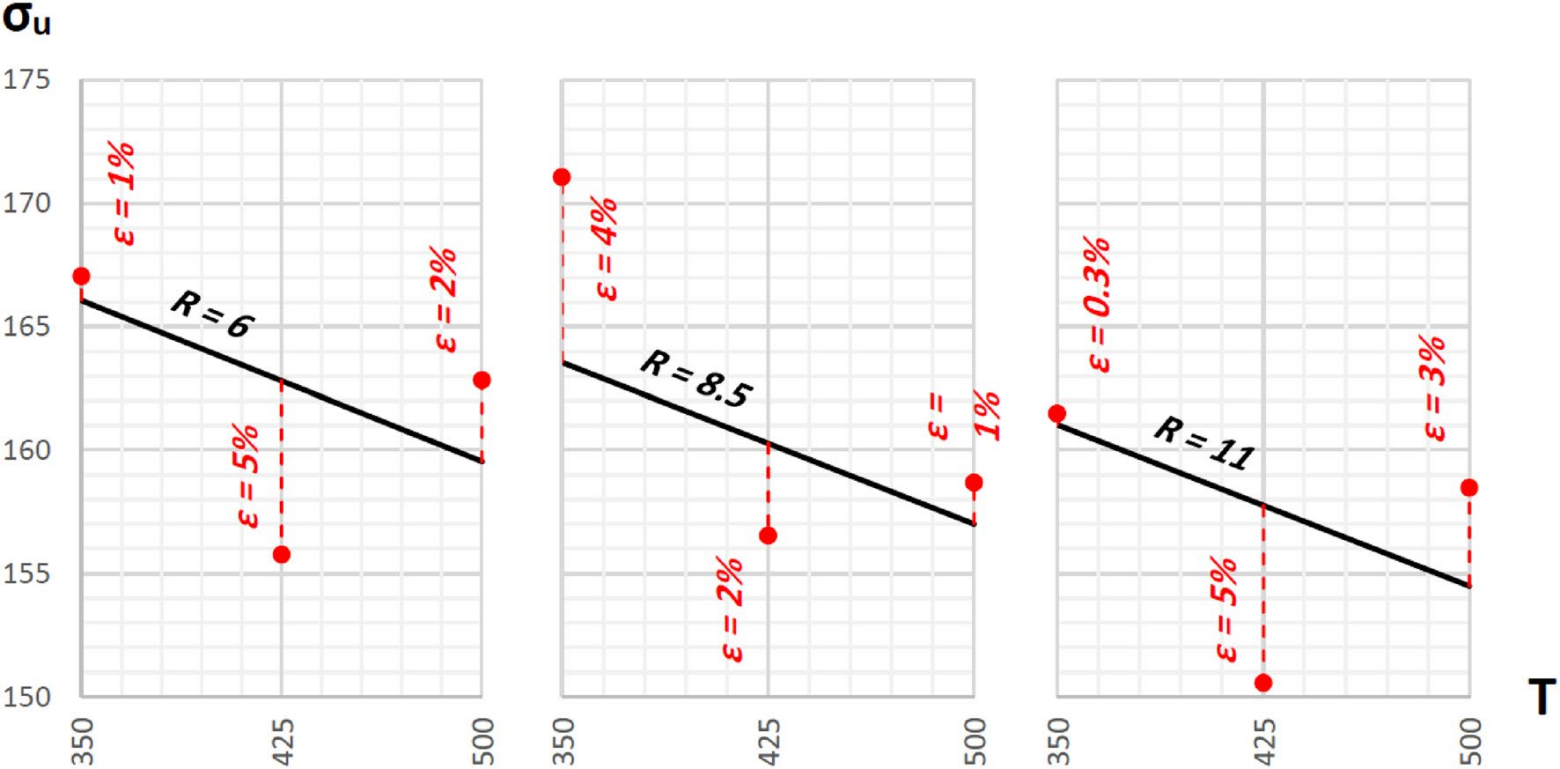
Regression lines representing theoretical ultimate strengths plotted against actual measured points and the errors between them (red dashed lines).

Following the abovementioned formulae, the linear regression equations (plotted in Figs. 8 and 9) for theoretical yield and ultimate strengths are:

**Table:** 

σ_y,th_ = 155.03 − 0.116T − 1.585R + ε, with R_σ,R_ = − 0.32, R_σ,T_ = − 0.71, R^2^ = 0.609	σ_u,th_ = 187.34 − 0.044T − 1.008R + ε, with R_σ,R_ = − 0.35, R_σ,T_ = − 0.46, R^2^ = 0.335

Both yield and ultimate tensile strength models exhibit negative relationships between the independent variables and the dependent variable, signifying that as either variable increases, the tensile strength tends to decrease. The second independent variable has a much stronger association with yield strength, as evidenced by its higher correlation coefficient, implying a clearer influence on its variation. Similarly, for ultimate tensile strength, the second variable shows a stronger association, although the overall correlation coefficients are weaker.

While the coefficient of determination suggests a moderate ability of the yield strength model to explain the dependent variable, there's still a significant portion of unexplained variance. This hints at potential limitations of the model or the presence of other contributing factors not included in the analysis. The lower coefficient of determination for the ultimate tensile strength model further emphasizes this, highlighting the need for further investigation into potentially missing factors or model refinement.

Figures 8 and 9 illustrate that the highest yield and ultimate tensile strengths, 105.06 MPa and 166.07 MPa, are optimally reached with an extrusion temperature of 350°C and a reduction ratio of 6.

In the previous section, ANOVA analysis revealed that both temperature and reduction ratio significantly affect ultimate strength, while only temperature impacts yield strength. Using this in the linear regression models, it is revealed that a 36% increase in reduction ratio combined with a 20% increase in temperature yielded a proportional decrease of 2% in ultimate strength. Similarly, a 20% increase in temperature alone resulted in a 9% proportional decrease in yield strength.

### Density

Every sample representing different extrusion conditions was evaluated using an analytical balance density determination kit. The outcomes of these evaluations are presented in Table 6. The table displays how the density of each specimen is slightly lower than the parent material, which is reasonable due to the possibility of voids and impurities during the compaction and extrusion processes.

**Table 6:** Density values of each extrudate representing every variable combination.

Sample #	0			1			2			3			4		
Ratio	–			11			11			8.5			8.5		
Temp	–			350			500			350			500		
Pos	A	0B	0C	1A	1B	1C	2A	2B	2C	3A	3B	3C	4A	4B	4C
Density	2.693	2.695	2.698	2.674	2.697	2.654	2.658	2.666	2.673	2.658	2.674	2.677	2.651	2.672	2.673
Avg. density	2.695*			2.675			2.666			2.670			2.665		

Sample #	5			6			8			9			10		
Ratio	6			6			11			6			8.5		
Temp	350			500			425			425			425		
Pos	5A	5B	5C	6A	6B	6C	8A	8B	8C	9A	9B	9C	10A	10B	10C
Density	2.631	2.657	2.661	2.679	2.661	2.642	2.652	2.677	2.658	2.693	2.668	2.666	2.668	2.670	2.664
Avg. density	2.650			2.661			2.662			2.676			2.667		

*Density value of the parent material

Two analytical methods were used to examine each value. The first approach was the ANOVA method, which was also used to investigate the tensile test outcomes. It aimed to determine the importance of each variable separately and its interaction with the variation between each value and that of the parent material. The second method employed was the linear multiple regression analysis, which involved calculating both correlation and determination coefficients (R & R^2^) to evaluate the strength, direction, and quality of the connection between the extrusion conditions (independent variables) and the density values. The results for each method are shown in Table 7.

**Table 7:** Density values analytical tests results—(A) Analysis of Variance (ANOVA) in two-factor experiment; (B) Linear multiple regression analysis.

	Sources of variation	MSR	Min. MSR
(A)	Among columns (ratio)	${{\text{MSR}}}_{{\text{c}}}=0.47$	F_0.05:2:18_ = 3.55
	Among rows (temp)	${{\text{MSR}}}_{{\text{r}}}=0.26$	F_0.05:2:18_ = 3.55
	Column-row interaction	${{\text{MSR}}}_{{\text{cr}}}=1.51$	F_0.05:4:18_ = 2.93
(B)	R_ρ,R_ = 0.29	R_ρ,T_ = -0.06	R^2^ = 0.089

According to the findings presented above, it can be concluded that neither the individual variables nor their interaction have a significant impact on the density value of the extruded material. This is because all MSR values are lower than their corresponding minimum values. Moreover, the correlation and determination coefficients did not approach either + 1 or − 1, indicating a weak or nonexistent linear relationship between the extrusion variables and the density values. This could be due to two potential reasons:

- Impurities: Variations in unseen trace elements, even within acceptable ranges, could influence density through differing atomic weights and packing efficiencies, potentially masking the subtle impact of extrusion parameters.
- Limited range of influence: If the extrusion parametric variations used in the study did not cause a large enough change in density, the ANOVA and regression analyses might not have had enough data to detect a statistically significant effect.

### Microstructure

**Figure 10:**
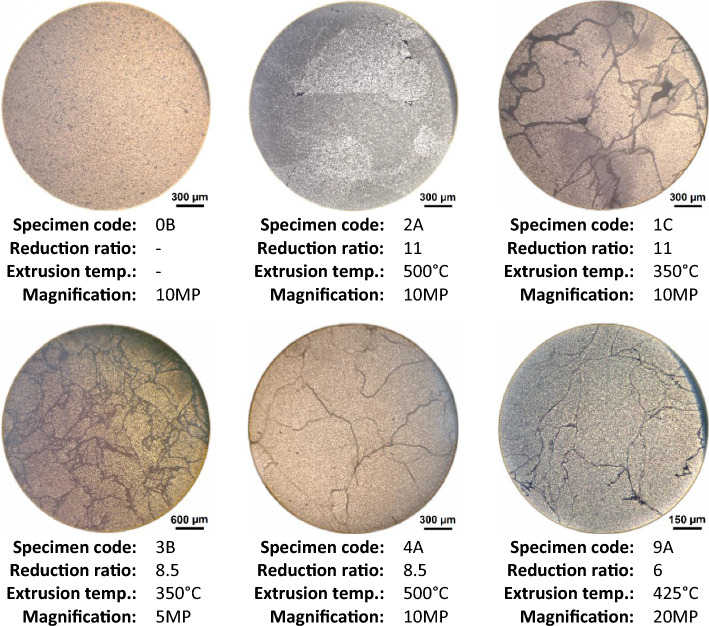
Microstructural images of different extruded samples at three magnification levels.

In the microstructure analysis phase, as shown in Fig. 10, it was clearly observed that as both reduction ratio and extrusion temperature increase, the sample’s grain size increases, while the grain boundaries and voids intensity decreases; giving a visually more similar structure to that of the parent material (Specimen code: 0B).

## Conclusion

This study aimed to determine the optimal operating and forming factors for recycled aluminum material, evaluate the effect of direct hot extrusion on improving the material properties, and identify the optimal conditions for the direct hot extrusion process. The experimental work included selecting the aluminum alloy material, conducting mechanical and metallurgical tests on the original material, extracting the chips using a selected operation, turning the raw material at different depths, compressing the chips into blocks, and then hot extruding the blocks into the desired cross-sectional shapes. The final product's properties were compared with those of the original material. Through investigation, the following conclusions have been made:

- Microstructural analysis showed that it would be beneficial to increase both extrusion reduction ratio and temperature (within the capabilities of the material and machines used) in order to obtain a product with a more stable and uniform structure.
- It has been discovered, through ANOVA analysis, increasing both extrusion reduction ratio and temperature would only be beneficial in case the recycled material is to be used in metal-forming applications.
- If the recycled material is to be used in applications which only require elastic deformation, the main focus should only be on increasing the extrusion temperature, as the yield strength of the recycled material is not significantly impacted by the extrusion reduction ratio.
- Implementing those findings into the linear multiple regression (LMR) models, it is found that increasing both reduction ratio (36%) and temperature (20%) leads to a slight decrease in ultimate strength (2%), while higher temperature alone (20%) reduces yield strength more significantly (9%).
- Based on LMR analysis, it was discovered that the optimum extrusion temperature and reduction ratio values to obtain the highest yield tensile strength (105.06 MPa) and ultimate tensile strength (166.07 MPa) are 350°C and 6, respectively.
- Based on both ANOVA and LMR analyses, it's clear that the extrusion temperature and reduction ratio, both individually and combined, do not significantly affect the material's extruded density. This is potentially due to the combined effects of subtle variations in trace element impurities and a limited range of influential change in density caused by the extrusion parameter values used in the study.
- Microstructure results show that high extrusion temperature and reduction ratio result in better mechanical properties, while tensile test results and ANOVA show that low extrusion temperature and reduction ratio result in better mechanical properties. Such contradiction can only be explained due to the fact that at high temperatures, the material has undergone a similar process to annealing, which led to decreasing its overall mechanical properties.

Overall, the study provides insights into the potential of direct hot extrusion as a method for recycling aluminum chips and identifies the optimal operating conditions for producing high-quality recycled aluminum material.

## Data Availability

The datasets used and/or analyzed during the current study available from the corresponding author on reasonable request.

## References

[1] Haase M, Tekkaya AE (2015). Cold extrusion of hot extruded aluminum chips. J. Mater. Process. Technol..

[2] Güley V, Güzel A, Jäger A, Ben Khalifa N, Tekkaya AE, Misiolek WZ (2013). Effect of die design on the welding quality during solid state recycling of AA6060 chips by hot extrusion. Mater. Sci. Eng. A..

[3] Baolong M, Xiaofei L, Zhongjun J, Jiefan J (2019). Recycle more, waste more? When recycling efforts increase resource consumption. J. Clean. Prod..

[4] Gronostajski JZ, Kaczmar JW, Marciniak H, Matuszak A (1997). Direct recycling of aluminium chips into extruded products. J. Mater. Process. Technol..

[5] Tekkaya AE, Schikorra M, Becker D, Biermann D, Hammer N, Pantke K (2009). Hot profile extrusion of AA-6060 aluminum chips. J. Mater. Process. Technol..

[6] Chiba R, Nakamura T, Kuroda M (2011). Solid-state recycling of aluminium alloy swarf through cold profile extrusion and cold rolling. J. Mater. Process. Technol..

[7] Zuo X, Chen X, Hu J (2023). Effect of extrusion temperature on microstructure and mechanical properties of solid-state recycled AA6063 aluminum alloy. Materials.

[8] Zhang J, Xu Y, Wang Z, Zhang L, Xu M (2021). Effect of preheating temperature on microstructure and mechanical properties of solid-state recycled AA5083 aluminum alloy. Metals.

[9] Wagdy B, Abd El-Wahab AA, El-gamasy R (2023). Statistical analysis of solid-state recycled aluminum alloy 2011 chips by hot extrusion. J. Eng. Sci. Military Technol..

[10] El-Habbas AM, Saleh HA, Azmi AM (2017). Solid-state recycling of aluminum alloy (AA-6061) chips via hot extrusion followed by equal channel angular pressing (ECAP). Egypt. Int. J. Eng. Sci. Technol..

[11] Wang J, Lin Y, Chang T, Lee S (2006). Recycling the magnesium alloy AZ91D in solid state. Mater. Transact..

[12] The Aluminum Association Registration Record Series: Teal Sheets; International Alloy Designations and Chemical Composition Limits for Wrought Aluminum and Wrought Aluminum Alloys (2015)

[13] Machining of aluminum and aluminum alloys. *ASM Handbook*. **16**, 761–804 (1989).

